# Plant Innate Immunity Multicomponent Model

**DOI:** 10.3389/fpls.2015.00987

**Published:** 2015-11-13

**Authors:** Giuseppe Andolfo, Maria R. Ercolano

**Affiliations:** Department of Agricultural Sciences, University of Naples ‘Federico II’Portici, Italy

**Keywords:** biotrophic, necrotrophic, IAC, IMC, phytohormone, plant defense, primary metabolism

## Abstract

Our understanding of plant–pathogen interactions is making rapid advances in order to address issues of global importance such as improving agricultural productivity and sustainable food security. Innate immunity has evolved in plants, resulting in a wide diversity of defense mechanisms adapted to specific threats. The postulated PTI/ETI model describes two perception layers of plant innate immune system, which belong to a first immunity component of defense response activation. To better describe the sophisticated defense system of plants, we propose a new model of plant immunity. This model considers the plant’s ability to distinguish the feeding behavior of their many foes, such as a second component that modulates innate immunity. This hypothesis provides a new viewpoint highlighting the relevance of hormone crosstalk and primary metabolism in regulating plant defense against the different behaviors of pathogens with the intention to stimulate further interest in this research area.

The PTI/ETI model postulates two forms of plant innate immunity, whereas most of evidences indicate the occurrence of an unique type. The basis of innate immunity in plants, as in the case of innate immunity in vertebrates, is mediated through a single overarching principle, the perception of signals of danger (Jones and Dangl, 2006). The evolutionary separation of innate immunity described in the PTI/ETI model, based on the perception of pathogen-specific molecular classes (PAMPs and effectors), is not sufficient to explain the modulation of resistance responses when both molecule types can trigger plant nonspecific immunity (Jones and Dangl, 2006; McDowell and Simon, 2008). In addition, there is often little effective resistance to necrotrophs that produce nonspecific toxins, cell wall degrading and defense suppressing enzymes, suggesting that these powerful virulence functions may override PTI and ETI processes (Heil and Land, 2014).

For plants, the perception of endogenous elicitors or Danger/Damage-Associated Molecular Patterns (DAMPs) may trigger signals of pathogen invasion similar to PAMPs/effectors as reported in others eukaryote organisms (Hein et al., 2009; Heil and Land, 2014). The responses triggered by DAMPs largely overlap with those activated by PAMPs. The surface-localized receptors (PRRs) perceive DAMPs and thus activate the resistance response. In plants, DAMPs can induce a set of basal responses such as indirect and direct antimicrobial effects (cell wall strengthening and anti-microbial agents) and also serve as signals (prime defense responses). Therefore, the defense activation may be considered as recognition of ‘non-self’ (PAMPs or effectors) or ‘altered-self’ (DAMPs) (Heil and Land, 2014).

PAMPs, DAMPs, and effectors are perceived by the plant as signals of danger that alert the defense system. Different methods of (pathogen) recognition are present in the extracellular space or in the cytoplasm of the host (Boller and Felix, 2009). The perception of all these signals appears to trigger the stereotypical defense program, albeit with kinetic and quantitative differences in induction (Wise et al., 2007). In their defense response, plants seem not to discriminate between PAMPs or DAMPs and effectors originating from bacteria, virus, fungi, or oomycetes. The response to effectors typically results in a hypersensitive response, whereas PAMPs or DAMPs do not normally cause cell death. However, this is not a general rule because some PAMPs could induce a hypersensitive response (Ron and Avni, 2004; Takemoto et al., 2005; Thomma et al., 2011), whereas some resistance genes provide protection without a hypersensitive response (Lee et al., 2006).

The pathogen recognition genes (Nibblers, PPRs) seem to be incapable of unequivocally distinguishing a specific pathogen by its feeding behavior in order to modulate a specific resistance response. They are involved in perception of pathogen invasion and alerting the non-specific immune system responses. Numerous cases have been reported in the literature in which the same *R-*gene confers resistance to more than one pathogen while different *R-*genes confer resistance against multiple pathogens (Tai et al., 1999; Zhao et al., 2005; Gururani et al., 2012). The innate immunity of vertebrates, also known as a non-specific immune system, defends the host from infection by other organisms in a non-specific manner.

In all stages of plant growth and development phytohormones play essential roles as signaling molecules that regulate cellular processes locally but also systemically (Loake and Grant, 2007; Bari and Jones, 2009). They also play a crucial role in the regulation of plant immune responses to microbial pathogens (Shah, 2003; von Essen et al., 2010). Similar to vertebrates, these hormones can act as immunomodulators, altering the sensitivity of the immune system, and act as mediators and regulators of immune processes (Schenk et al., 2000). The balance of hormonal crosstalk strongly influences the outcome of plant–pathogen interactions, including the establishment of effective immunity. Rapid adaption to threats from the biotic environment is regulated by an enormous regulatory network of interconnect signal pathways. Several studies have reported that plant–pathogen interaction, involving biotrophic pathogens, requires salicylic acid (SA) signaling modulation, whereas a combination of jasmonic acid (JA) and ethylene (ET) signaling modulation is required in interactions with necrotrophic pathogens (Glazebrook, 2005). However, the new emerging picture indicates that complex crosstalk among different classes of hormones might modulate the disease resistance, with outcomes dependent on the pathogen lifestyles and the genetic constitution of the host (Mur et al., 2006; Robert-Seilaniantz et al., 2011; Kazan and Lyons, 2014).

Many phytopathogens are able to manipulate plant hormone signaling pathways to counteract plant defense responses. Tactics frequently employed by plant pathogens involve hijacking, evading, or disrupting hormone signaling pathways and/or crosstalk. This is achieved mechanistically via pathogen-derived molecules (effectors), which target components of phytohormone signaling pathways in the host plant. Pathogens also use “phytohormone mimics,” molecules that structurally and/or functionally resemble phytohormones or phytohormone signaling components, to trick the host into behaving inappropriately. In turn, plants have adopted innovative strategies and diverse mechanisms to neutralize these attacks, often relying on elaborate signaling networks regulated by phytohormones (Bolton, 2009).

The attempted infection of biotrophs and necrotrophs can activate plant immune responses, which include complex histological, cellular, biochemical, and molecular events that the pathogen proliferation or disease spread is limited. Lifestyle, infection strategy and host defense responses vary greatly between the two pathogen classes. The typology of damage signals release from the injured host tissue (DAMPs and GLVs) can also help to better regulate host response (Scala et al., 2013; Heil and Land, 2014). The damaged-self recognitions (PRRs mediated) inform the host on tissue disrupted and contribute to trigger both JA- and SA-mediated responses (Scala et al., 2013; Heil and Land, 2014). Positive feedback loops, characteristic of DAMP-mediated signaling, serve to prime the same cell or the surrounding tissue for future injury or infection (Heil and Land, 2014).

It has been suggested that during plant–pathogen interactions the role of primary metabolism is to support the cellular energy requirements for plant defense response which establishes a favorable energy balance for defense (Bilgin et al., 2010; Kangasjarvi et al., 2012). Consistent with these notions, it appears that the up-regulation of defense-related pathways is compensated by the down-regulation of genes involved in photosynthesis as well as pathogen-derived elicitors (Andolfo et al., 2014; Rojas et al., 2014). Recently, several studies on the role of primary metabolic pathways (photosynthesis, assimilate partitioning, and source–sink regulation) in different plant–pathogen interactions focused the attention on the role of primary metabolism in regulating the plant defense response after pathogen attack (López-Gresa et al., 2010). Metabolic feedback regulation triggered by pathogenetic factors and mediated by the suppression of photosynthesis and sugar signals are indicated as the most reliable system since pathways are reprogrammed thanks to the metabolic effects induced by pathogen. The different lifestyles of biotrophic and necrotrophic pathogens are due to the need to complete their life cycle on living or dead tissues, respectively. The comparison of the different changes induced by biotrophic and necrotrophic pathogens revealed the complexity and divergence of the responses of plant primary metabolic pathway (Rolland et al., 2006; Duan et al., 2013). Thus demonstrating, that the plant defense is preceded and facilitated by a fundamental shift of primary metabolism (Scharte et al., 2005).

Consistent with this notion, it is possible to conceive a well-articulated model in which specific interactions, derived by host and pathogen action overlapping spaces, generate different defense responses (**Figure 1**) (Walley et al., 2007). In this schema, the action spaces of resistance plant (green triangle) and of biotrophic and necrotrophic pathogens (blue and red rectangles, respectively) are indicated. The intersections among action areas identify three plant–pathogen interaction areas, two of which are specific to pathogen lifestyle (small black triangles) and one is common (violet circle) to both the lifestyle-dependent pathogen interactions. In the violet circle the Immunity Activation Component (IAC), composed by PRRs-Triggered Signaling (PTS), and Nibblers-Triggered Signaling (NTS), it is independent of the pathogen feeding behavior and actives the plant defense-signaling. IAC makes it possible to discern a biotic interaction from a physical or chemical form of abiotic stress (Dodds and Rathjen, 2010). The transmembrane and cytoplasmic receptors play a key role since they act as sentinels for the recognition of pathogens in cellular specific areas (cytoplasmic and extracellular spaces). The recognition components contribute to making immediate the host response, but it is not sufficient to explain the fine-tuning defense signaling by the plant during the interaction with biotrophic or necrotrophic pathogens (Dodds and Rathjen, 2010).

**FIGURE 1:**
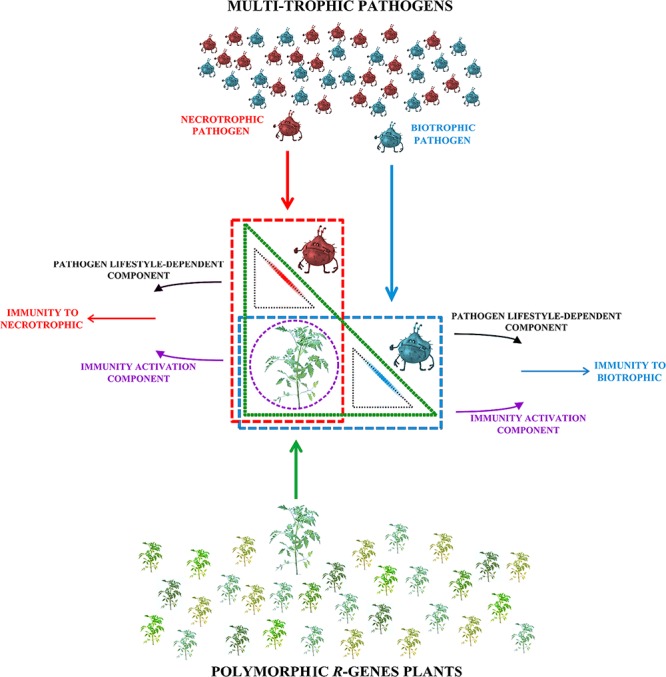
**Two different resistance directions are supposed to be activated in a multi-trophic interaction.** Once a plant containing a *R*-gene (different green shades) comes in contact with biotrophic or necrotrophic pathogens, only an incompatible interaction will activate the plant resistance. In this scheme, the interaction spaces of plant resistance (green triangle) and of biotrophic and necrotrophic pathogens (blue and red rectangles, respectively), are indicated. The intersections of interaction spaces identify three plant–pathogen interaction areas: two are pathogen lifestyle-related (small black triangles) and one is common (violet circle). The synergic effect of immunity activation and of pathogen lifestyle-dependent components result in plant immunity to biotrophic or necrotrophic pathogens.

Once a plant comes in contact with a biotrophic or necrotrophic pathogen, only an incompatible interaction will create an exchange of information necessary to activate the plant resistance. The feeding behavior of the pathogen also influences the activation of a second immunity component that is responsible for the differential modulation of the resistance, which will drive the resistance in right direction (Rolland et al., 2006). In **Figure 1**, two small black triangles depict the overlapping areas between host (green triangle) and biotrophic/necrotrophic pathogens (blue and red rectangles, respectively), that initiate the IAC converging in the pathogen-specific plant immunity.

Based on the observation of plant pathogen lifestyle-dependent interaction (schema in **Figure 1**), we propose new insights that contribute to a model of plant innate immune system (**Figure 2**). Our circular model schematically illustrates the key points of two components (activation and modulation) plant immunity and the resultant of their combination. In the circular model, the plant–pathogen interaction could be synthetized in three phase: (1) interaction, (2) activation, and modulation (3) effective resistance. During the interaction stage when the pathogen (fungi, virus, bacteria, and oomycetes) interacts with the host, two principal effects are detected: (A) modifications of virulence factor targets (Bolouri-Moghaddam and Van den Ende, 2012) and (B) specific alterations of primary plant metabolism (López-Gresa et al., 2010; Duan et al., 2013). These two biological responses determine the transition to the activation stage of resistance. Direct and indirect perception of virulence factors, mediated by pathogen recognition genes (NB-LRRs; RLKs; RLPs) triggered the plant defense-signaling (Wise et al., 2007; McDowell and Simon, 2008; Boller and Felix, 2009).

**FIGURE 2:**
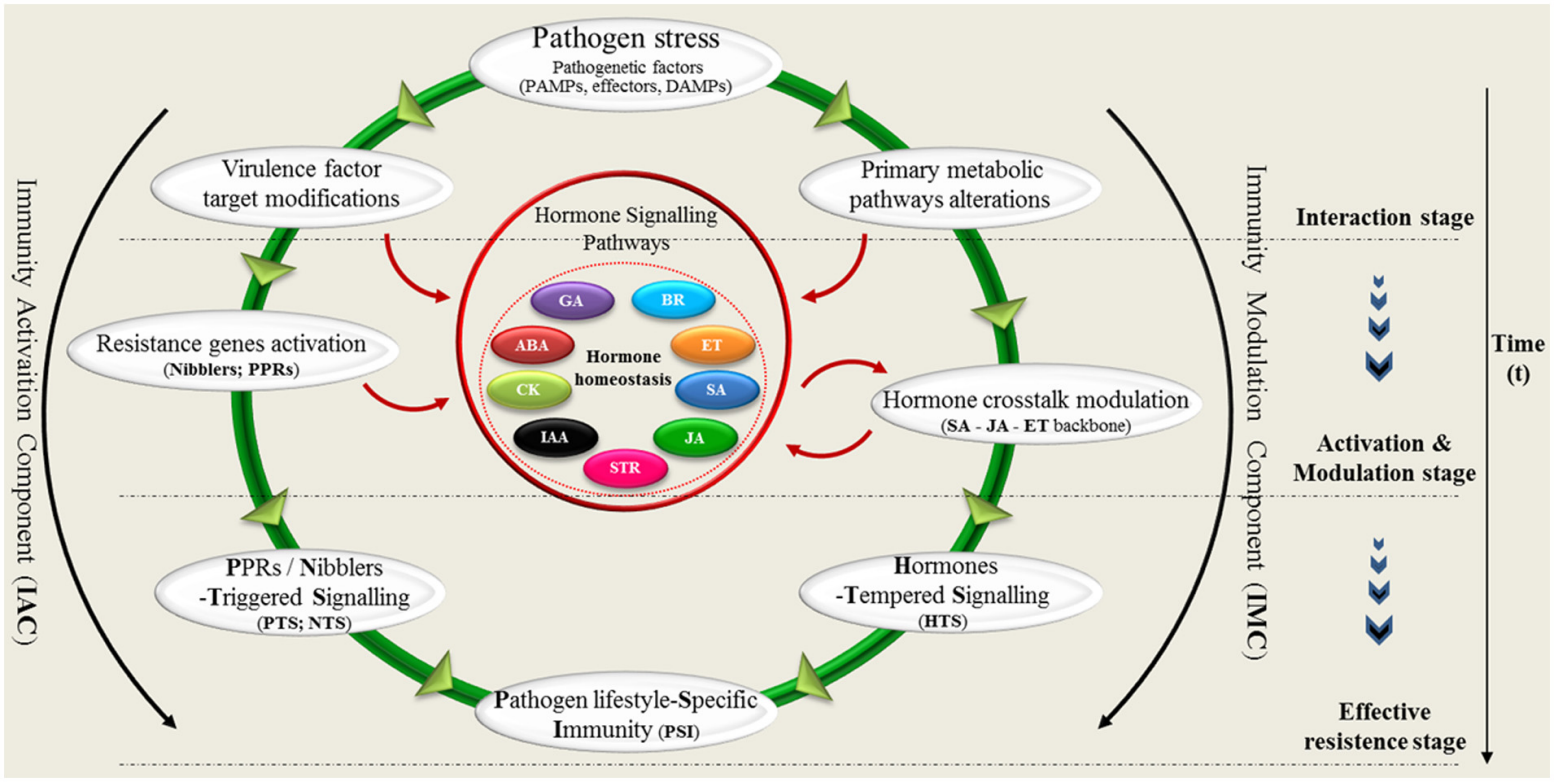
**The circular model.** The model schematically shows the key points of activation and modulation of plant immunity. Plant resistance mechanism of an incompatible interaction might be divided into three phases: (1) interaction, (2) activation/modulation, and (3) effective resistance (immunity). During the interaction stage, two principal effects are detected: (A) modifications of virulence factor targets and (B) specific alterations of primary plant metabolism. In the activation stage: the modifications of virulence factor targets induce the Nibblers Triggered Signaling (NTS) or PPRs Triggered Signaling (PTS), mediated by *R*-genes activation. These metabolic alterations induce a feedback regulation of primary metabolic pathways resulting in a Hormone Tempered Resistance (HTR). In the effective resistance stage, the NTS/PTS, and the HTR converge to confer a resistance specific to the lifestyle of pathogen (Pathogen lifestyle-Specific Resistance, PSR).

In plants, sugar signals are generated by photosynthesis and carbon metabolism in source and sink tissues to modulate growth, development, and stress responses. During the recognition phase metabolic alterations induced from pathogen attack initiate feedback regulation of plant primary metabolism, mediated by sugar signals and genes involved in photosynthesis and chlorophyll biosynthesis (Scharte et al., 2005; Rolland et al., 2006). The various alterations of primary metabolism induced by the feeding behavior of microbial pathogens generate a calibrated hormone response. Several lines of evidence illustrate the intimate cross-talk of JA, gibberellins (GA), auxins (IAA), cytokines (CK), ET, and sugar signaling pathways (Audenaert et al., 2002; Li et al., 2007). Interestingly, there is extensive crosstalk between sugar-specific signaling pathways and abscisic acid (ABA) signaling pathways. ABA antagonizes SA (Asselbergh et al., 2008) but synergizes with JA (Pieterse et al., 2009), suggesting a pivotal role for ABA between these two pathways. It has been shown that the cross-talk among GA, JA, ABA, and sucrose in a complex signaling network can modulate immune response, and notably, sucrose signaling seems to be a primary and essential component in this network (Roitsch, 1999). Feedback regulation of metabolism stimulates hormone signaling crosstalk that modulates the resistance response (Scharte et al., 2005). The metabolic shift from source to sink further enhances the plant hormone signaling, and the expression of defense-related genes (Scharte et al., 2005; Scala et al., 2013).

It has been clearly shown that the production of three major phytohormones (JA, ET, and SA) mediates the defense response to different pathogen lifestyles (Glazebrook, 2005; Loake and Grant, 2007; Bari and Jones, 2009). In our circular model the hormone-regulated signaling defense pathways play a central role in plant immunity modulation. The plant defense system is fine-tuned and carefully modulated for responses to the different feeding behaviors of microbial pathogens. Recently, brassinosteroids (BR) and strigolactones (STR) have been shown to interact antagonistically or synergistically with the SA-JA-ET backbone of the plant innate immune signaling network (**Figure 2**) (Glazebrook, 2005). In conclusion, the specific lifestyle of pathogens requires a specific response. In the effective resistance stage, the IAC and the Immunity Modulation Component (IMC) converge in a unique response of resistance specific to the lifestyle of pathogen (Pathogen lifestyle-Specific Immunity, PSI). Our IAC/IMC model presents a schematic representation of plant innate immune components in which plant hormones play a leading role in determining the outcome. The plant possesses an internal and external receptor repertoire that can activate prompt pathogen recognition. Plant global awareness requires a metabolic response directly bearing on the established interaction. More studies are necessary to identify additional components involved in defense responses as well as a detailed characterization of the mechanisms underlying such responses.

## Author Contributions

Conceived and designed the model and was centrally involved in manuscript writing: GA. Elucidated the meaning and revised the paper: MRE. Both authors read and approved the final manuscript.

## Conflict of Interest Statement

The authors declare that the research was conducted in the absence of any commercial or financial relationships that could be construed as a potential conflict of interest.
